# Conformation of G-quadruplex Controlled by Click Reaction

**DOI:** 10.3390/molecules25184339

**Published:** 2020-09-22

**Authors:** Chao-Da Xiao, Zhi-Yong He, Chuan-Xin Guo, Xiang-Chun Shen, Yan Xu

**Affiliations:** 1 State Key Laboratory of Functions and Applications of Medicinal Plants, School of Pharmaceutical Sciences, Guizhou Medical University, University Town, Guian New District, Guiyang 550025, China; sxc@gmc.edu.cn; 2 Medical Sciences, Faculty of Medicine, University of Miyazaki, 5200 Kihara, Kiyo-take, Miyazaki 889-1692, Japan; chiyuu_ga@med.miyazaki-u.ac.jp; 3 Nucleic Acid Division, Shanghai Cell Therapy Group Co. Ltd., Jiading, Shanghai 201805, China; guocx@shcell.com

**Keywords:** G-quadruplex, conformation change, click reaction, NMR, circular dichroism

## Abstract

G-quadruplexes are non-canonical four stranded secondary structures possessing great biological importance. Controlling G-quadruplex conformation for further regulating biological processes is both exciting and challenging. In this study, we described a method for regulating G-quadruplex conformation by click chemistry for the first time. 8-ethynyl-2′-deoxyguanosine was synthesized and incorporated into a 12-nt telomere DNA sequence. Such a sequence, at first, formed mixed parallel/anti-parallel G-quadruplexes, while it changed to anti-parallel after reaction with azidobenzene. Meanwhile, the click reaction can give the sequence intense fluorescence.

## 1. Introduction

G-quadruplexes are higher-order DNA and RNA structures found in nucleic acid sequences that are rich in guanine residues [1,2,3]. Four guanine bases that are hydrogen-bonded via Hoogsteen pairings form a G-quartet. G-quartets linked by loops can stack upon each other, forming a G-quadruplex. Different topologies of G-quadruplexes including anti-parallel, parallel, and hybrid G-quadruplex have been observed in several x-ray and NMR structures [4,5,6,7,8,9,10,11,12,13]. The G-quadruplex has been proven widely as the essential biological regulators. Bioinformatics analysis shows as many as 3000 different 5′-untranslated regions (UTRs) of mRNAs may contain the putative quadruplex sequences, which has been proven to exert important regulating roles in post-transcriptional gene expression [14]. Furthermore, studies show that they can regulate telomere length, form telomeric heterochromatin, and protect telomere [15,16,17,18].

The local conformation of G-quadruplex is suggested to correlate with biological function regulation. For example, binding of hnRNPA1 to telomeric RNA is structure dependent. HnRNPA1 can bind to telomere RNA G-quadruplex with a loop structure rather than the G-quadruplex without a loop [19,20]. Developing a convenient method to regulate biological processes by controlling the conformation of the G-quadruplex would be meaningful [21,22].

Cu(I) catalyzed Huisgen 1,3-dipolar cycloaddition (CuAAC) is one of the popular click chemistry. As it possesses operational simplicity, specificity, orthogonality, modularity, and biocompatibility, the CuAAC reaction gains enormous popularity in the field of chemical biology. [23,24,25,26] For example, the CuAAC reaction was successfully applied to localize a bioactive compound inside living cells. [27] Herein, we developed a novel method utilizing the CuAAC reaction to control the guanine nucleosides’ glycosidic conformation, then changing the whole conformation of the G-quadruplex.

As for the G-quadruplex formation, the glycosidic conformation of the guanine nucleosides in the G-quartet is essential for the G-quadruplex topology. In parallel G-quadruplex, all guanine nucleosides possess the *anti* conformation for the glycosidic bond orientation. While, in the anti-parallel G-quadruplex, half guanine nucleosides are in the *anti* conformation and half in the *syn* conformation [28]. Controlling the conformation of the guanine nucleosides can induce the G-quadruplex topology, changing between the parallel and anti-parallel.

**Figure 1:**
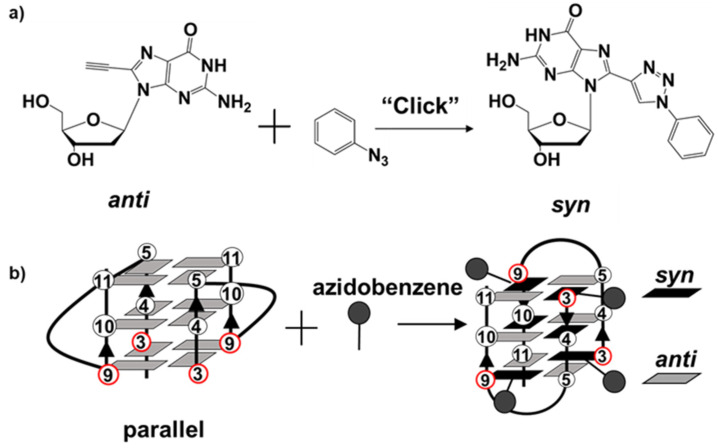
(**a**) Click reaction controls the glycosidic conformation of modified guanine nucleoside. (**b**) G-quadruplex topology changed by manipulating the glycosidic conformation of modified guanine nucleoside through click reaction.

Incorporation of a substituent group at the C8 position of dG usually causes a steric hindrance between the 8-substituent and the ribose ring, resulting in adopting the *syn* conformation. Thus, we tried to use click reactions to introduce the substituent group at the C8 position of dG to control the conformation (Figure 1a,b).

## 2. Results

**Scheme 1:**
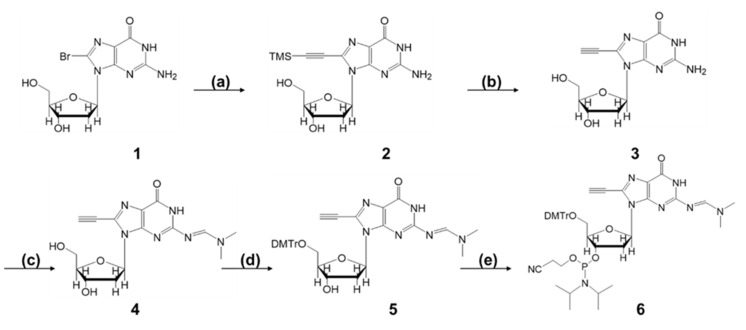
Reagents and conditions: (**a**) Trimethylsilyl acetylene, Tetrakis(triphenylphosphine)palladium, Triethylamine, *N,N*-Dimethylformamide, 50 °C, 3 h; (**b**) Tetrabutylammonium fluoride, Tetrahydrofuran, rt, 2 h; (**c**) *N,N*-dimethylformamide diethylacetal, methanol, 55 °C, 3 h; (**d**) 4,4′-Dimethoxytriphenylmethyl chloride, Triethylamine, 4-(Dimethylamino)pyridine, Pyridine, rt, overnight; (**e**) *N,N*-diisopropylchlorophosphoramidite, Triethylamine, rt, 15 min.

**Figure 2:**
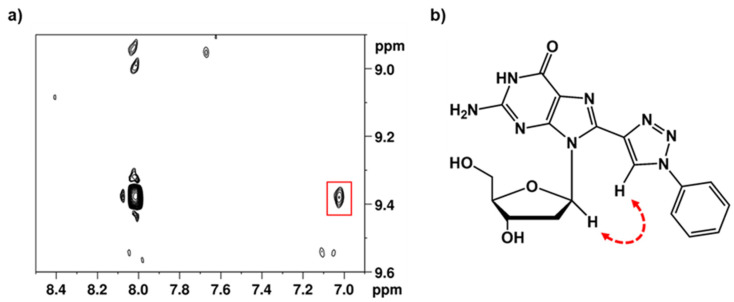
(**a**) NOESY spectrum of 8^et^dG click reaction product. Cross peak between H in the triazole ring and H1′ is in the red box. (**b**) Schematic representation of *syn* glycosidic conformation of 8^et^dG click reaction product with arrows indicating the NOEs.

We synthesized 8-ethynyl-2′-deoxyguanosine (8^et^dG) (Scheme 1 and Figures S1–S8, ESI†). The synthesis of the 8-ethynyl-2′-deoxyguanosine (8^et^dG) begins with 8-bromo-2′-deoxyguanosine, and the preparation of 8^et^dG-containing oligonucleotides was carried out by phosphonamidite chemistry. The Nuclear Overhauser Effect Spectroscopy (NOESY) was used to check the glycosidic conformation of 8^et^dG before the click reaction. Results showed that the H1′ of 8^et^dG gave NOEs to H4′. Meanwhile, a cross peak between H5′ and the proton in the 8-ethynyl group was also observed. Such results provided the evidence that 8^et^dG adopted *anti* glycosidic conformation and the C3′-endo conformation of sugar (Figure S9). While, after the azide–alkyne cycloaddition reaction with azidobenzene, the product showed that the H in the triazole ring gave Nuclear Overhauser Effects (NOEs) to the H1′, which indicated that the click product adopted the *syn* glycosidic conformation (Figure 2a,b and Figure S10, ESI†). The above results indicated the alkynyl substitution did not alter the deoxyguanosine glycosidic conformation, while such a conformation can be changed by the click reaction. Therefore, it is possible to use 8^et^dG for changing the G-quadruplex topology though the click reaction.

Previous study showed that the 12-nt telomeric DNA (TAGGGTTAGGGT, ODN-N) can adopt both parallel and anti-parallel G-quadruplex structures in K^+^-containing solution [29]. In the anti-parallel G-quadruplex structure, the dGs (③, ⑨) in the core GGG stretch were in the *syn* conformation, while *anti* conformation in parallel G-quadruplex (Figure 1b). Therefore, inducing the guanine nucleosides’ glycosidic conformation changing of the ③, ⑨ dGs can change the overall topology of ODN-N, which may be the ideal sequence for testing the strategy above. Next, we substituted the dGs (③, ⑨) in ODN-N with the 8^et^dG generating the modified sequences ODN-3 and ODN-9, and tried to manipulate the parallel part of the 12-nt telomeric DNA G-quadruplex.

**Figure 3:**
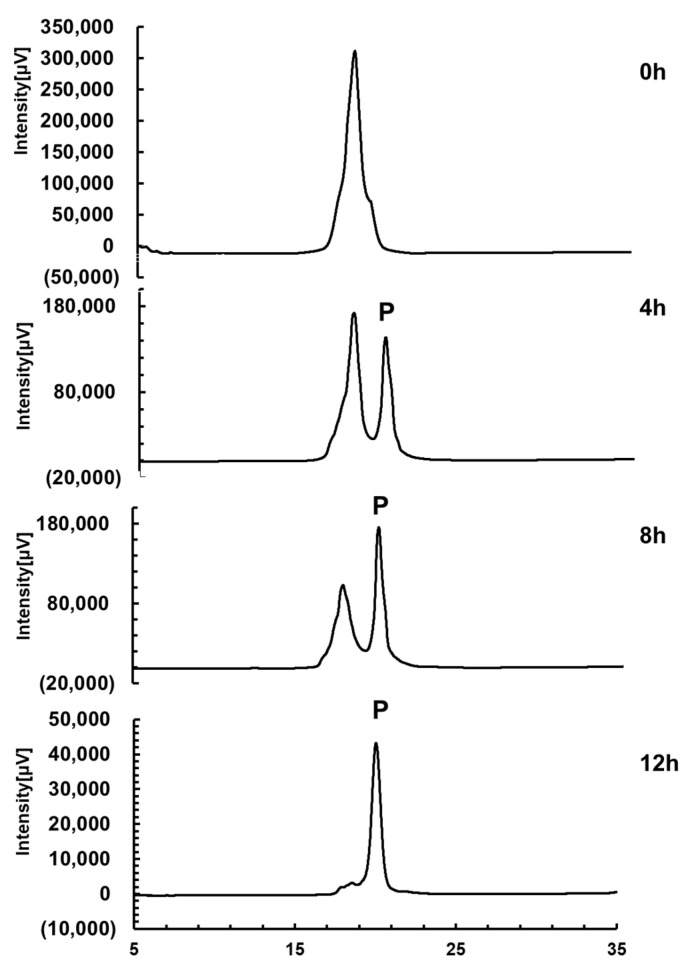
HPLC analyses of the ODN-N reaction with the CuAAC reaction solution at the indicted time where peak “P” is the product.

We initially checked the click reaction with the 8^et^dG incorporated into the DNA sequence. Briefly, ODN-9 was subjected to the CuAAC reaction solution containing azidobenzene, Tris[[1-(3-hydroxypropyl)-1H-1,2,3-triazol-4-yl]methyl]amine, sodium ascorbate, and CuSO_4_·5H_2_O. High Performance Liquid Chromatography (HPLC) and Matrix-Assisted Laser Desorption/ Ionization Time Of Flight Mass Spectrometry (MALDI-TOF-MS) were used to monitor the reaction. As shown in Figure 3, the ODN-9 slowly reacted with azidobenzene. HPLC results showed that after four hours, the click product amount/unreacted ODN-9 was 42/58, after 8 h it was 72/28, and after 12 h, ALL ODN-9 exhibited the formation of product (“P”) having the mass (3897.344 Da) (Figure S11, ESI†). The slow reaction may be due to the steric hindrance induced by the G-quadruplex, which may prevent the azidobenzene from entering the core GGG stem. Next, we verified the conformation changing of the G-quadruplex through the click reaction.

Consistent with previous study, the Circular Dichroism (CD) spectrum of ODN-N showed two positive peaks at 295 nm and 265 nm, which clearly indicated that a mixture of parallel and anti-parallel G-quadruplexes was formed (Figure S12, ESI†) [29,30,31]. Comparing the CD spectrum of ODN-3 and ODN-9, ODN-9 showed similar CD features with ODN-N, which indicated that the substitution of dG ⑨ with 8^et^dG did not perturb the characteristic of the native sequence forming both the parallel/anti-parallel G-quadruplex, whereas the ODN-3 showed different CD features with a broad peak at 295 nm and a small shoulder around 265 nm. Then, ODN-9 was used to evaluate the conformation changing of the G-quadruplex by the click reaction.

**Figure 4:**
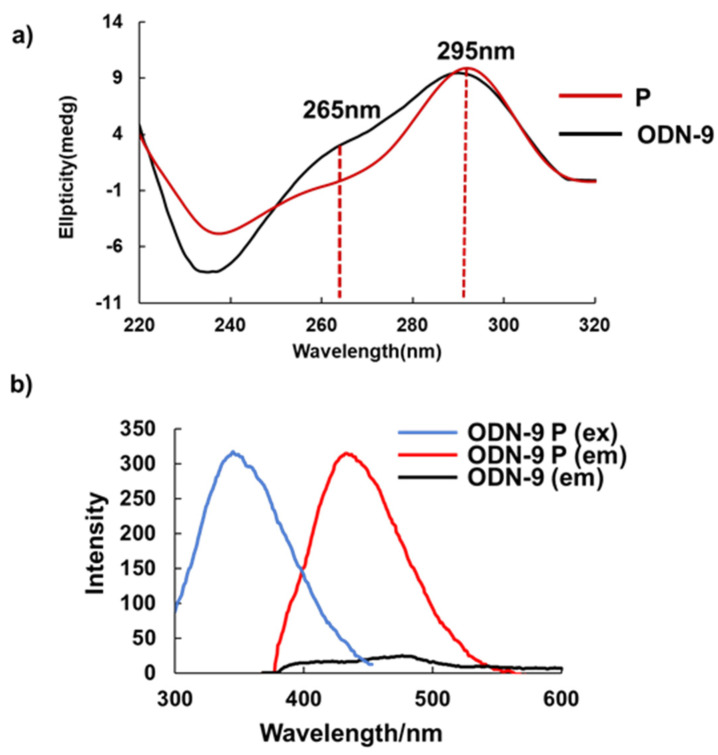
(**a**) CD spectrum of unreacted and click reaction product of ODN-9. (**b**) Fluorescence spectra for modified oligonucleotides. ODN-9 P (em): the emission spectrum for click reaction product of ODN-9; ODN-9 (em): emission spectrum for the ODN-9 unreacted sequence; ODN-9 P (ex), excitation spectrum (345 nm).

After the ODN-9 was mixed with the CuAAC reaction solution, the CD result of the click reaction product showed only a positive peak at 295 nm, indicating the parallel G-quadruplex was converted to anti-parallel (Figure 4a).

Biological imaging is always important for the study of biology. For example, chromosome imaging is essential for chromosome analysis and genetic diagnostics. Traditional methods using dye molecules do not form covalent bonds with chromosome. Therefore, it is difficult to monitor chromosome dynamics. The click reaction can selectively form a covalent bond between the chromosomal DNA and fluorescent molecules, which may be an ideal method for chromosome dynamics analysis [23]. Recently, we used 5-ethynyl-2′-deoxyuridine and direct visualization of the chromosome behavior in cell division through the azide–alkyne click reaction [23]. The click reaction has also been applied in the labeling of myoglobin, bovine serum albumin, and other proteins [23,32,33,34]. Next, we checked the fluorescence spectra of the click reaction product to investigate whether the 8^et^dG would be used for imaging application. We found that the triazole product by the cycloaddition reaction gave the ODN-9 an intense fluorescence. Formation of the triazole ring by azide–alkyne cycloaddition acts as a π-conjugator, linking the guanine quartet and electron-rich benzene moiety, which results in a strong fluorescence. The fluorescence spectrum of the click reaction product exhibited an emission band at 445 nm, while the ODN-9 unreacted sequence showed no emission (Figure 4b).

In conclusion, we synthesized 8^et^dG, and incorporated it into the telomeric DNA sequence. Based on various chemical approaches, we proved the incorporating 8^et^dG could convert the G-quadruplex from parallel conformation to anti-parallel though click reaction. Although the parallel G-quadruplex may be expected to be dominated in cells, proteins or other factors interact with the anti-parallel G-quadruplexes. Controlling these structures to play an essential biological role would be very interesting. ODN-9 can be endowed with fluorescence through the click reaction, which further conformed the reaction between the modified sequence and azidobenzene. Such a result also indicates that further study should investigate the potential of 8^et^dG for the application in biomolecular labelling.

## 3. Materials and Methods

### 3.1. Sample Preparation

The synthesis of the 8-ethynyl-2′-deoxyguanosine-containing DNAs were carried out by phosphoramidite chemistry. All DNAs were synthesized on the 1 μmol scale with an automatic DNA/RNA synthesizer (Nihon Techno Service Co. Ltd., Ushiku, Japan). After automated synthesis, the oligonucleotides were detached from the support and deprotected according to the manufacturer’s protocol. All oligonucleotides were purified by reverse phase-HPLC (JASCO).

### 3.2. CD Experiments

CD spectra were measured using an AVIV model 430 CD spectrophotometer. Samples were prepared by heating the oligonucleotides at 90 °C for 5 min and gradually cooling them to room temperature. Solutions for CD spectra were prepared as 0.3 mL oligonucleotides at 0.01 mM concentrations in the presence of 100 mM KCl and 10 mM Tris buffer (pH 6.8) and at 25 °C.

### 3.3. Click Reaction

To 20 µL Tris(3-hydroxypropyltriazolylmethyl)amin ligand (0.7 mM as final concentration), sodium ascorbate (1 mM), CuSO_4_·5H_2_O (100 μM), and azidobenzene (10 μM) were added sequentially to prepare the CuAAC reaction solution. ODNs solution (1 μM in water) was added to the CuAAC reaction solution and the reaction mixture was kept at room temperature for the corresponding time. After completion of the reaction, the solution was subjected to Micro Bio-Spin™ Size Exclusion Spin column for preliminary purification. Click reaction product solution was further analyzed by RP-HPLC.

For the click reaction of 8^et^dG, to 1 mL 8^et^dG solution (50 μM as final concentration, dissolved in methanol), Tris(3-hydroxypropyltriazolylmethyl)amin ligand (3 μM as final concentration), sodium ascorbate (5.5 μM), CuSO_4_·5H_2_O (1 μM), and azidobenzene (75 μM) were added sequentially. The solution was kept for 1 h at room temperature. The reaction mixture was purified by a middle pressure liquid chromatography (MPLC).

### 3.4. HPLC Experiment

HPLC analysis used an analytical column (InertSustainSwift, C18 5 µm 250 × 6.0 mm, Torrance, CA, USA) and a JASCO HPLC system. A gradient of 2.5% to 22.5% acetonitrile was applied over 36 min at 1 mL/min in 0.1 M triethylammonium acetate (TEAA, pH 7.4). Peaks were collected, and lyophilized to dryness. All purified samples were analyzed using MALDI mass spectroscopy.

### 3.5. Fluorescent Spectra Measurement

Fluorescent spectra were measured using an Agilent model Cary Eclipse spectrofluorometer. The spectra were recorded using a 1-cm path-length cell. For each sample, at least two spectrum scans were accumulated over a wavelength range from 300–650 nm.

## Supplementary Materials

The following are available online. Figures S1–S6: ^1^H-NMR spectra of synthesized compounds, Figure S7: ^31^P NMR spectrum of compound, Figure S8: High-resolution mass spectra (HRMS) of all synthesized compounds, Figure S9: NOESY spectrum of 8^et^dG, Figure S10: NOESY spectrum of 8^et^dG click reaction product, Figure S11: MALDI-TOF MS of ODNs used in this study, Figure S12: CD spectra of ODN-N(a);ODN-9(b);ODN-3(c).
