# Supplementary material for: Conformation of G-quadruplex Controlled by Click Reaction
Source: Molecules. 2020 Sep 22;25(18):4339. doi: 10.3390/molecules25184339 (PMC7570587; doi:10.3390/molecules25184339)
Supplement: Supplementary file 1 [file molecules-25-04339-s001.pdf]

molecules

Supplementary Materials

Conformation of G-quadruplex controlled by click reaction

Chao-Da Xiao [1,*], Zhi-Yong He [2], Chuan-Xin Guo [3], Xiang-Chun Shen [1] and Yan Xu [2,*]

[1] State Key Laboratory of Functions and Applications of Medicinal Plants, School of Pharmaceutical Sciences, Guizhou Medical University, University Town, Guian New District, Guizhou 550025, China; xiaobujiao@163.com (C.-D.X.); shenxiangchun@126.com (X.-C. S.)
[2] Medical Sciences, Faculty of Medicine, University of Miyazaki, 5200 Kihara, Kiyo-take, Miyazaki 889-1692, Japan; chiyuu_ga@med.miyazaki-u.ac.jp (Z.-Y.H.)
[3] Nucleic Acid Division, Shanghai Cell therapy Group Co.,Ltd. ,Jiading, Shanghai 201805, China;guocx@shcell.com (C.-X.G)
* Correspondence: xiaobujiao@163.com; Tel.: +86-0851-88416160 (C.-D.X.); xuyan@med.miyazaki-u.ac.jp; Tel.: +81-985-85-0993 (Y.X.)

General

^{1}H-NMR and ^{31}P-NMR spectra were recorded on a BRUKER (AV-400M) magnetic resonance spectrometer. DMSO-d_6 and $CDCl_3$ were used as the solvents. ^{1}H spectra chemical shifts (δ) are reported in parts per million (ppm) referenced to residual protonated solvent peak (DMSO-d_6, δ = 2.50, $CDCl_3$, δ = 7.26). Coupling constants (J) values are given in hertz (Hz). Signal patterns are indicated as br (broad), s (singlet), d (doublet), t (triplet), sept (septet), m (multiplet). All reagents were purchased from Aldrich, TCI (Tokyo Chemical Industry) or Wako (Wako Pure Chemical Industries). Thin layer chromatography (TLC) was performed using TLC Silica gel 60 F_{254} (Merck). Compounds were visualized using a UV lamp (254 nm) or staining with a potassium permanganate solution. Silica gel (Wakogel® C-300, 200-325 mesh) was used for column chromatography. Purification of products was also performed on a middle pressure liquid chromatography (MPLC) systems (EPCLC-AI-580S, Yamazen Corporation) equipped with silica gel column (Hi-Flash Column, Yamazen Corporation). High-resolution mass spectra (HRMS) were recorded by electrospray ionization (ESI) on an Exactive Orbitrap mass spectrometer instrument (Thermo Scientific). The reagents for DNA synthesis were purchased from Glen Research. Mass spectra of oligodeoxynucleotides were determined with a MALDI-TOF MS (BRUKER AUTOFLEX, negative mode) with ammonium citrate dibasic and 3-hydroxy-2-pyridinecarboxylic acid as matrix. The synthesis of 8-ethynyl-2′-deoxyguanosine phosphoramadite **6** was modified from procedures described by Shinohara et al[1]. 8-Bromo-2′-deoxyguanosine **1** was purchased from TCI (B4002, Tokyo Chemical Industry)

Procedure for the synthesis of modified nucleoside

8. -(Trimethylsilylethynyl)-2′-deoxyguanosine (2)

8-Bromo-2'-deoxyguanosine (**1**, 5.0 g, 14.4 mmol) was dissolved in dry DMF (100 ml). Trimethylsilylacetylene (3.2 ml, 22.0 mmol), $Pd(PPh_3)_4$ (4.9 g, 4.2 mmol), CuI (965.4 mg, 5.1 mmol) and Et_3N (11.4 ml) were added. The reaction mixture was stirred at 50 °C for 3 h under argon. After the reaction, mixture was evaporated. The residue was purified by silica gel column chromatography to give the compound **2** (3.4 g, yield is 65 %). ^{1}H-NMR (DMSO-d_6, 400 MHz) δ 10.82(s, 1H), 6.58(br, 2H), 6.23 (t, J = 6.8 Hz, 1H), 5.26 (d, J = 4 Hz, 1H), 4.86 (t, J = 6 Hz, 1H), 4.35 (ddd, J = 3.2, 3.4, 3.3 Hz, 1H), 3.78 (m, 1H), 3.62 (m, 1H), 3.01 (m, 1H), 2.11 (m, 1H), 0.27 (s, 9H); HRMS (ESI) for $C_{15}H_{21}O_4N_5Si$ $[M-H]^-$: Calcd. 363.1357; Found. 363.1365.

8. -ethynyl-2′-deoxyguanosine (3)

Compond **2** (3.4 g, 9.4 mmol) was dissolved in dry THF (100 ml). Tetrabutylammonium fluoride (12 ml, 12mmol) were added to the reaction mixture under argon and the solution was stirred at room temperature for 2 h. Product was collected by filtering and washed with chloroform to give the target compound **3** (2.1g, yield is 76 %).^{1}H NMR (DMSO-d_6, 400 MHz) δ 10.88 (br, 1H), 6.58 (br, 2H), 6.25 (dd, J = 6.5, 6.6 Hz, 1H), 5.28 (d, J = 4.1 Hz, 1H), 4.90 (dd, J = 5.8, 5.9Hz, 1H), 4.79 (s, 1H), 4.37 (m, 1H), 3.80 (ddd, J = 2.8, 5.6, 5.7 Hz, 1H), 3.62 (ddd, J = 5.7, 5.9, 12.0Hz, 1H), 3.51 (ddd, J = 5.6, 5.8, 12.0 Hz, 1H), 3.07 (m, 1H), 2.11 (ddd, J = 2.8, 6.5, 13.2 Hz, 1H) ; HRMS (ESI) for $C_{12}H_{13}O_4N_5$ $[M{-}H]^-$: Calcd. 291.0962; Found. 291.0971.

N^2-N′,N′-dimethylformamidine-8-ethynyl-2′-deoxyguanosine (4)

Compound **3** (0.6 g, 2.0 mmol) dissolved in methanol (15 ml) was added *N,N*-dimethylformamide diethylacetal (4.6 ml), and the solution was stirred at 55 ^{o}C for 3 h. After evaporation of the solvent, the residue was purified by silica gel column chromatography ($CHCl_3$/MeOH=15:1) to afford compound **4**(0.5 g, yield is 70 %). ^{1}H NMR (DMSO-d_6, 400 MHz) δ11.55 (br, 1H), 8.51 (s, 1H), 6.36 (dd, J = 4.4, 2.4 Hz, 1H), 5.33 (d, J = 4.2 Hz, 1H), 4.84 (m, 2H), 4.44 (ddd, J = 8, 3.2, 3.6 Hz 1H), 3.82 (dd, J = 5.2, 3.2 Hz, 1H), 3.04 (m, 7H), 2.15 (m, 1H); HRMS (ESI) for $C_{15}H_{18}O_4N_6$ $[M{-}H]^-$: Calcd. 346.1384; Found. 346.1382.

N^2-N′,N′-dimethylformamidine-5′-O-dimethoxytrityl-8-ethynyl-2′-deoxyguanosine (5)

The compound **4** (0.5 g 1.4 mmol) was co-evaporated with pyridine (10mL) three times and suspended in 10 mL anhydrous pyridine. 4,4′-Dimethoxytrityl chloride (0.8 g, 4.5 mmol), triethylamine (0.4 mL, 2.5 mmol), and 4-dimethylaminopyridine (5.1 mg, 48.2 umol) were added to the mixture. After it stood overnight, TLC (CH_2Cl_2 : CH_3OH = 10 : 1) showed complete reaction. The reaction was cooled in an ice bath, and 50 mL of aqueous 5 % $NaHCO_3$ solution was added. The mixture was extracted twice with 10 mL of dichloromethane. The organic layer was dried over Na_2SO_4, and concentrated *in vacuo*. The residue was purified by silica gel column chromatography to give the compound **5** (0.6 g, yield was 62 %). ^{1}H NMR (DMSO-d_6, 400 MHz) δ 11.52 (br, 1H), 8.32 (s, 1H), 7.28 (d, J = 8.0 Hz, 2H), 7.16 (m, 7H), 6.76 (dd, J=8.0, 7.8 Hz, 4H), 6.37 (t, J = 8.0, Hz 1H), 5.36 (d, J =4.0 Hz, 1H), 4.75 (s, 1H), 4.56 (m, 1H), 3.91 (br, 1H), 3.72 (s, 6H), 3.24 (dd, J=12, 4.0 Hz, 1H), 3.12 (m, 2H), 3.02 (s, 6H), 2.26 (br, 1H); HRMS (ESI) for $C_{36}H_{36}O_6N_6$ $[M{-}H]^-$: Calcd. 648.2690; Found. 648.2695.

N^2-N′,N′-dimethylformamidine-5′-O-dimethoxytrityl-8-ethynyl-2′-deoxyguanosine phosphoramidite (6)

The compound **5** (0.6 g, 0.87 mmol) was co-evaporated with anhydrous acetonitrile (10 mL) and anhydrous dichloromethane (2 mL) three times, followed by suspension in anhydrous acetonitrile (20 mL). Next, *N,N*-diisopropylethylamine (0.6 mL, 3.9 mmol) and 2-cyanoethyldiisopropylchlorophosphoramidite (0.5 mL, 2.9 mmol) were added and left for 15 min at room temperature. The reaction mixture was worked up with an aqueous 5 % $NaHCO_3$ solution (10 mL), and the mixture was extracted three times (10 mL) with dichloromethane containing 1% triethylamine. The organic layer was dried over Na_2SO_4, and concentrated *in vacuo*. The reaction mixture was purified by a middle pressure liquid chromatography (MPLC) in *n*-hexane containing 1 % triethylamine with a gradient formed by ethyl acetate to give the compound **6** (0.6 g, yield was 80 %). ^{1}H NMR ($CDCl_3$, TMS, 400 MHz) δ 8.42 (s, 1H), 7.38 (d, J =4 Hz, 2H), 7.20 (m, 4H), 6.74 (m, 4H), 6.49 (t, J = 7.2, 1H), 4.95 (br, 1H), 4.84 (s, 1H), 4.22 (m, 1H), 3.77 (m, 8H), 3.26(m, 2H), 3.06 (s, 2H), 3.00 (s, 6H) 2.66 (t, J=6Hz, 2H), 2.48 (m, J=6Hz, 2H); HRMS (ESI) for $C_{45}H_{53}O_7N_8P$ $[M{-}H]^-$: Calcd.849.3973; Found. 849.3864.

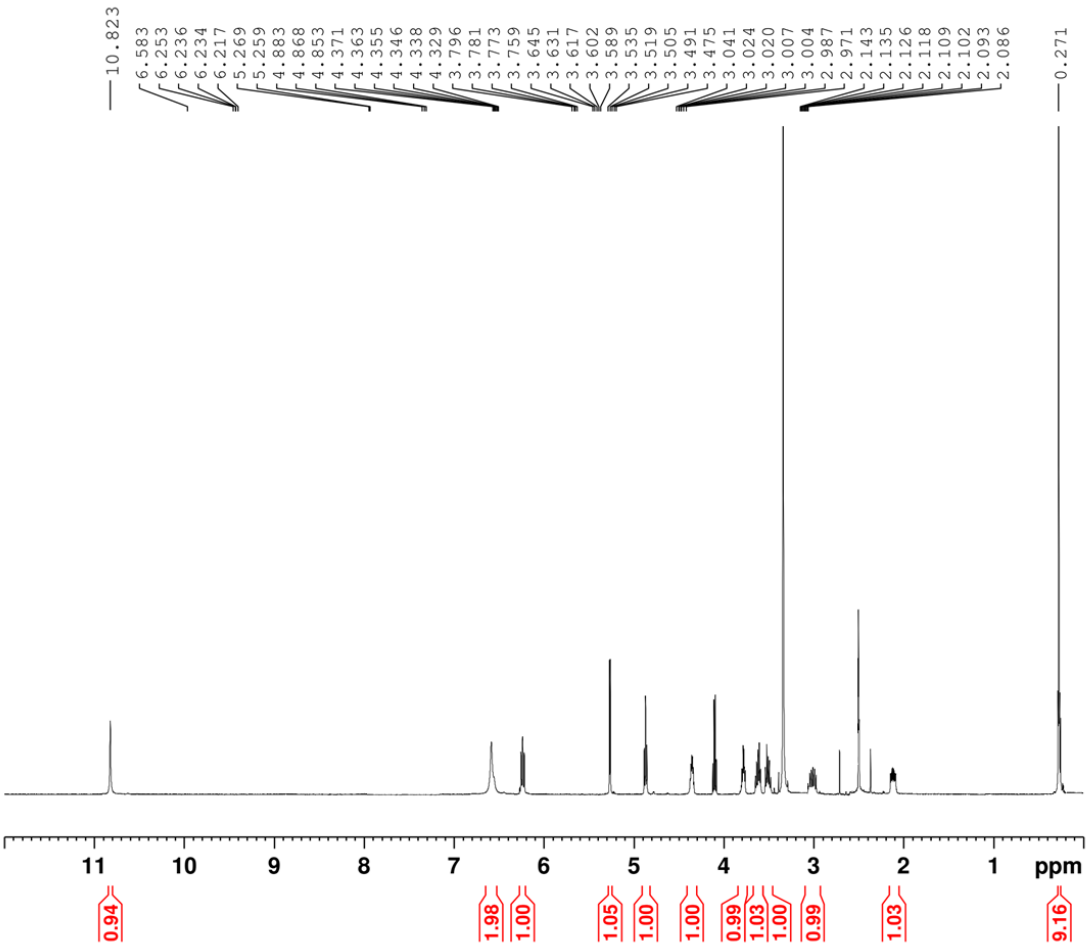

Figure S1. ^{1}H NMR spectrum of compound **2**.

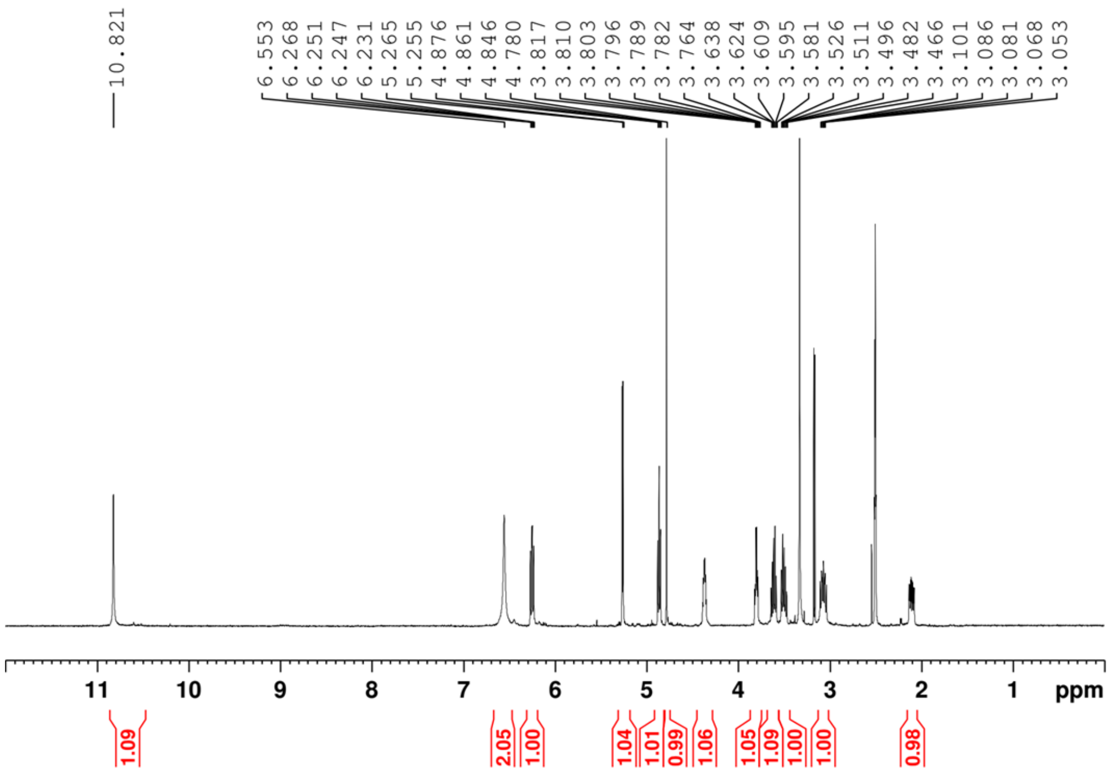

Figure S2. ^{1}H NMR spectrum of compound **3**.

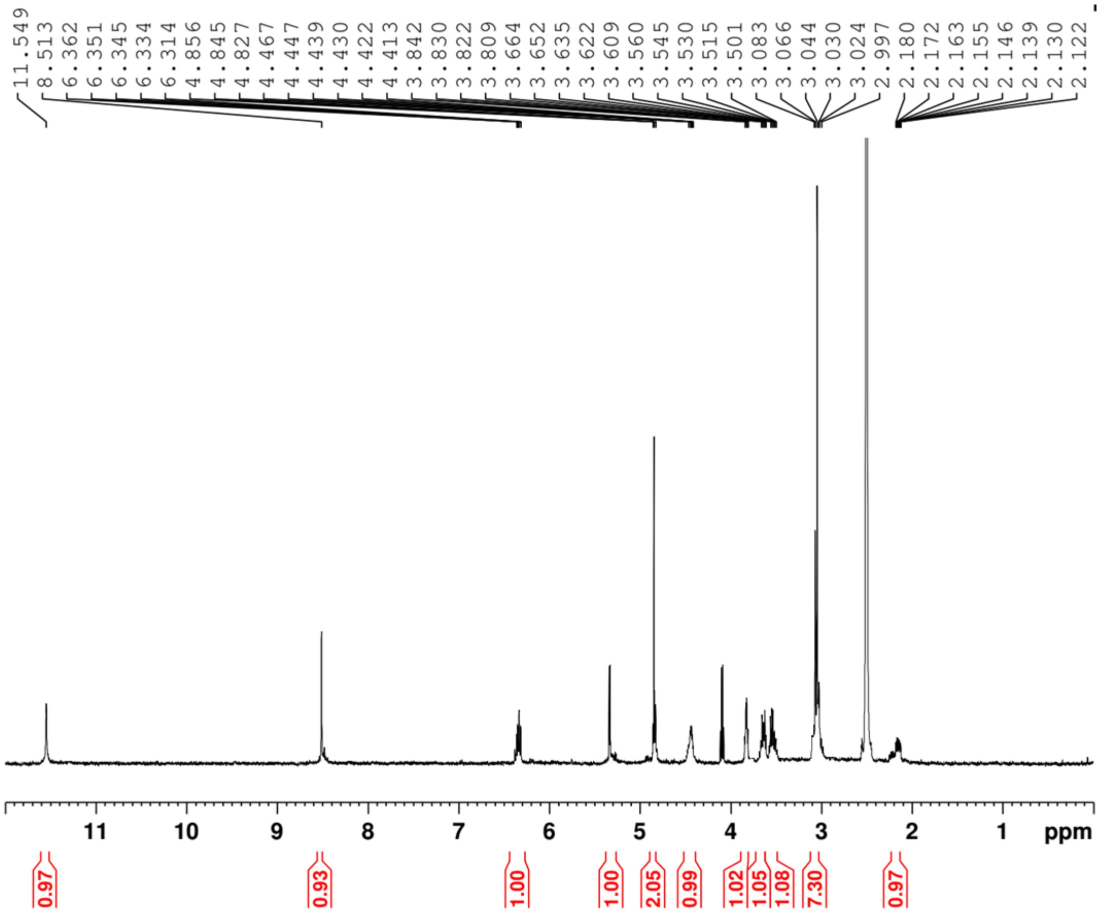

Figure S3. ^{1}H NMR spectrum of compound **4**.

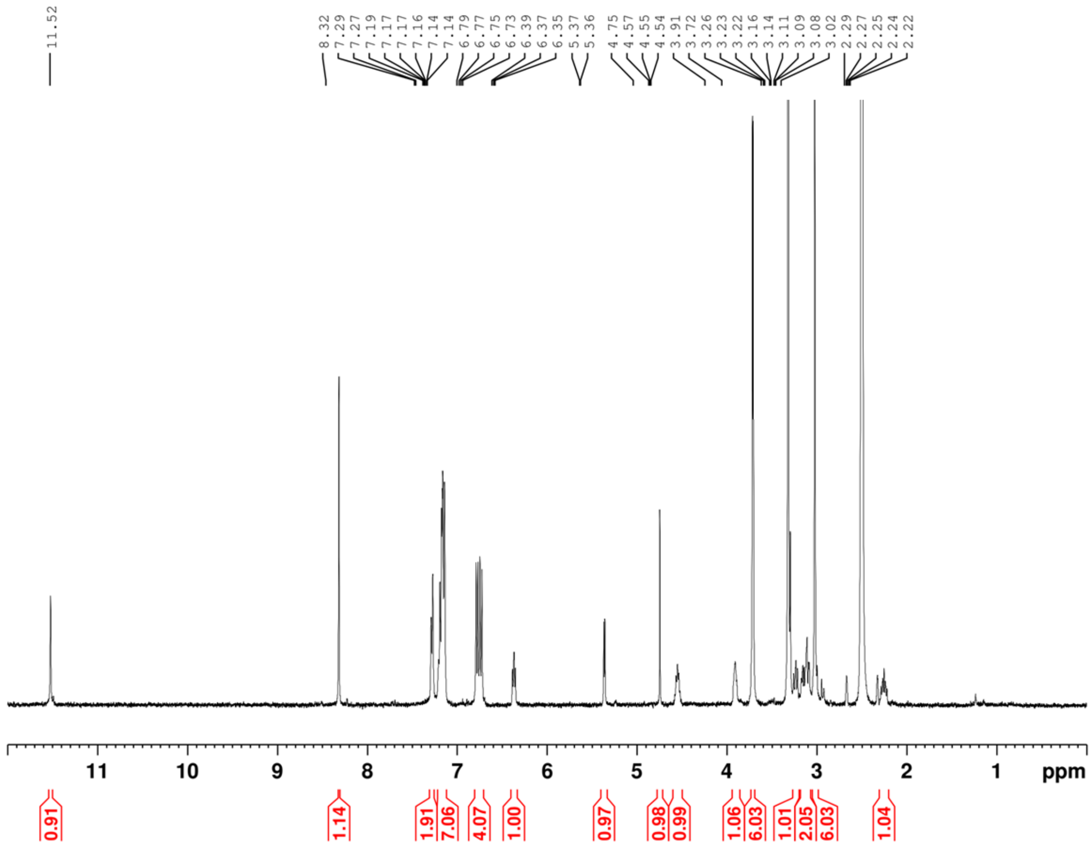

Figure S5. ^{1}H NMR spectrum of compound **5.**

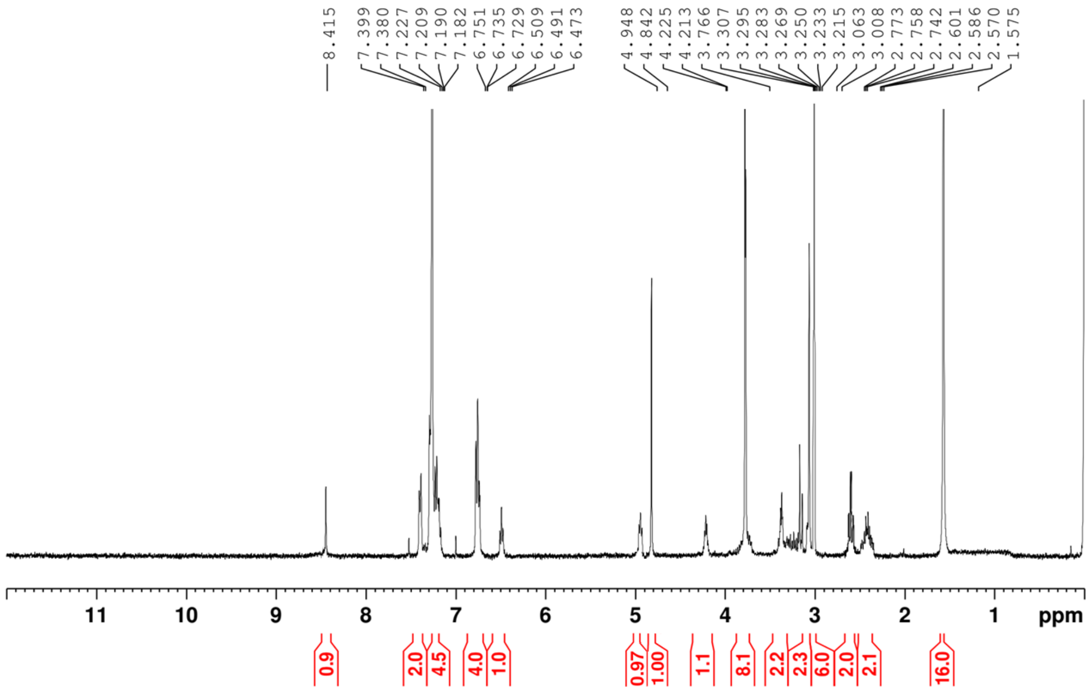

Figure S6. ^{1}H NMR spectrum of compound **6.**

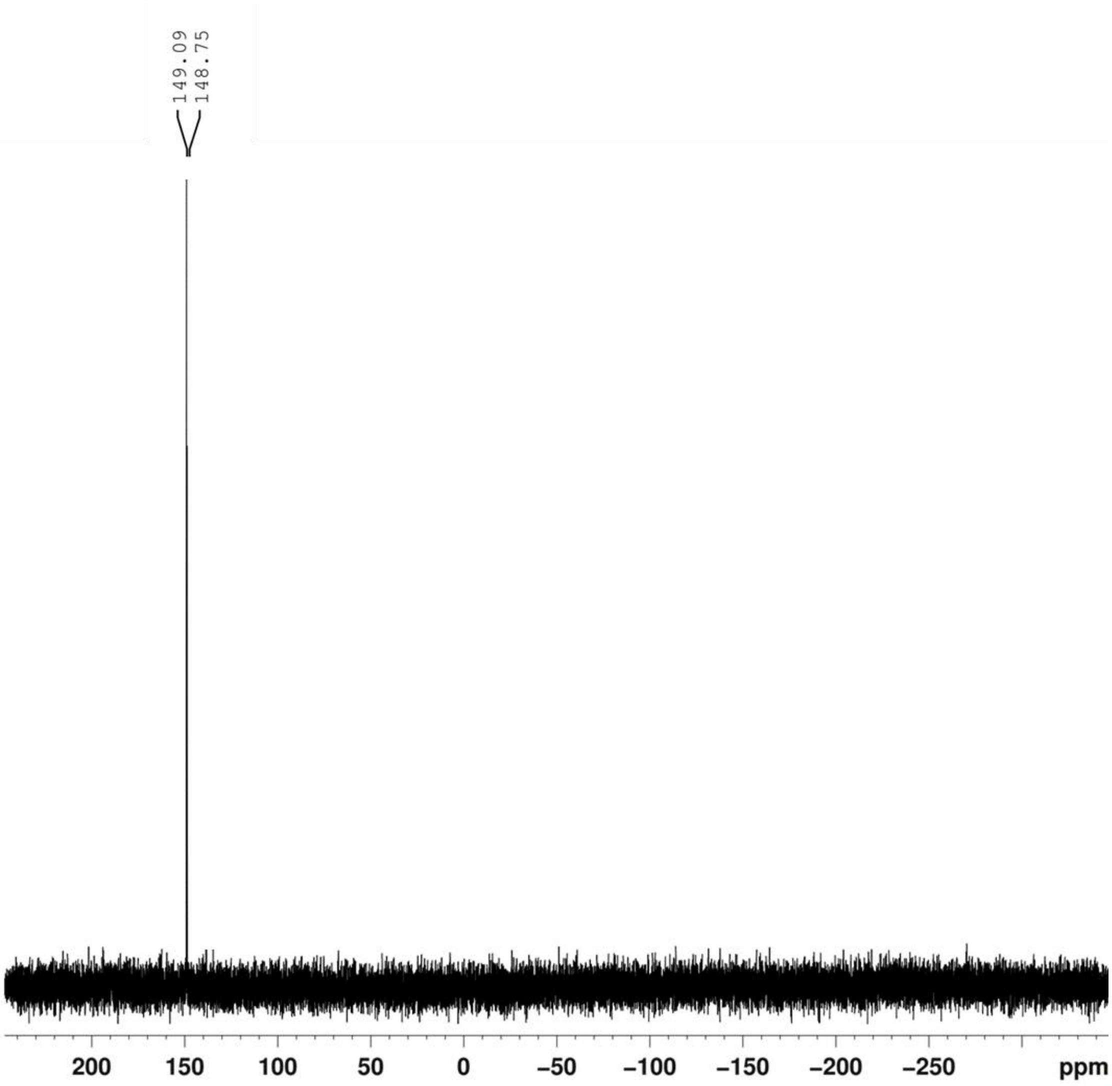

Figure S7. ^{31}P NMR spectrum of compound **6**.

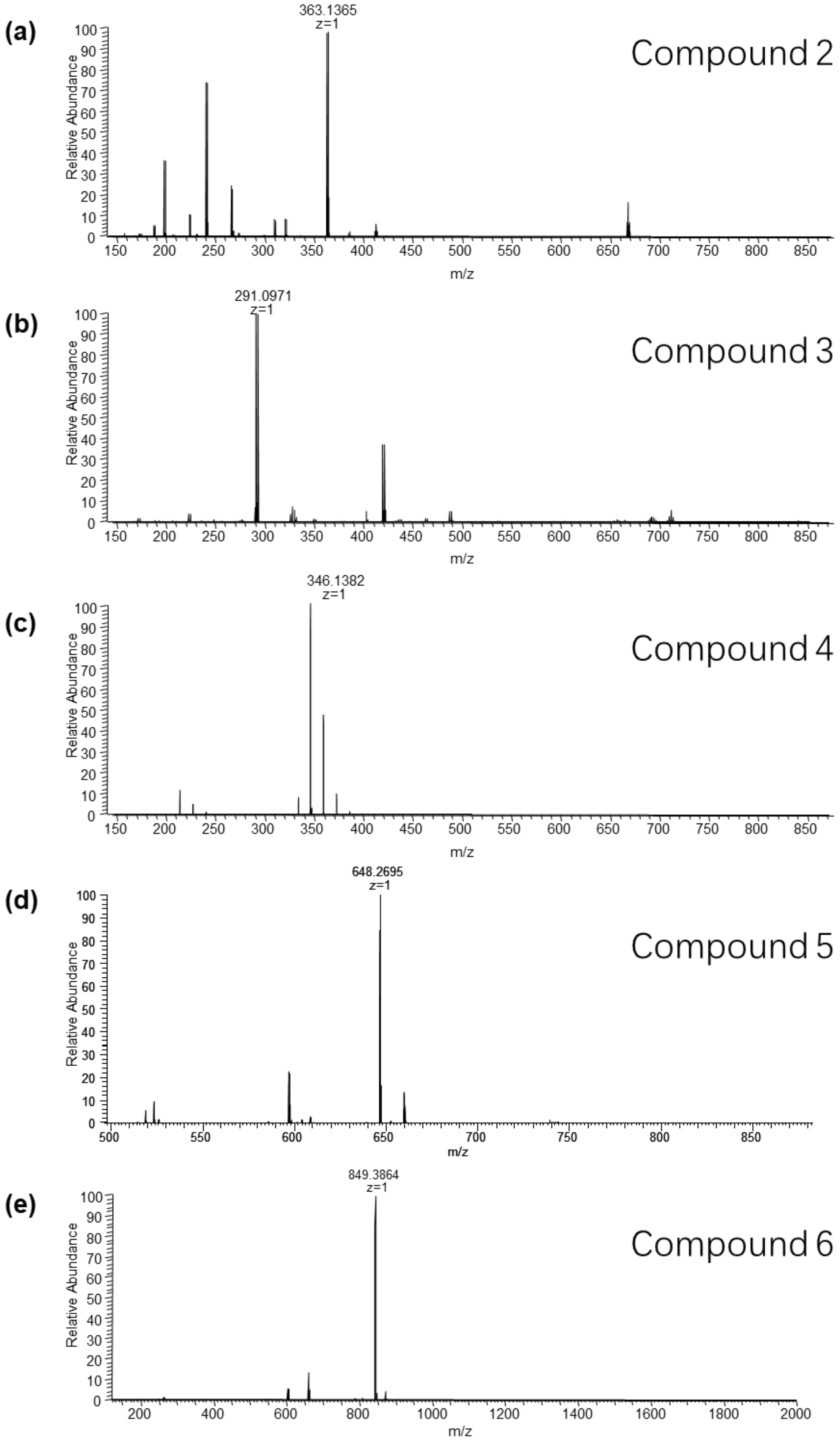

Figure S8. High-resolution mass spectra (HRMS) of all compounds used in this study.

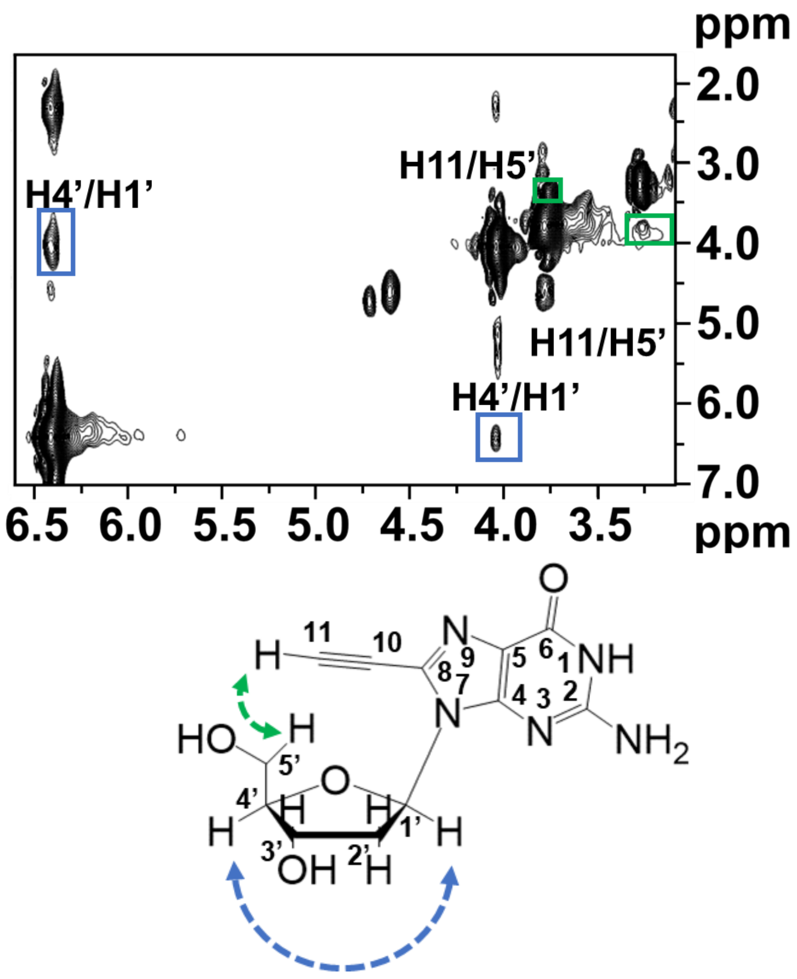

Figure S9 NOESY spectrum of 8^{et}dG. cross peak between H11 and H5′ is in the green box, cross peak between H1′ and H4′ is in blue box. Schematic representation of anti-glycosidic conformation of 8^{et}dG with arrows indicating the NOEs.

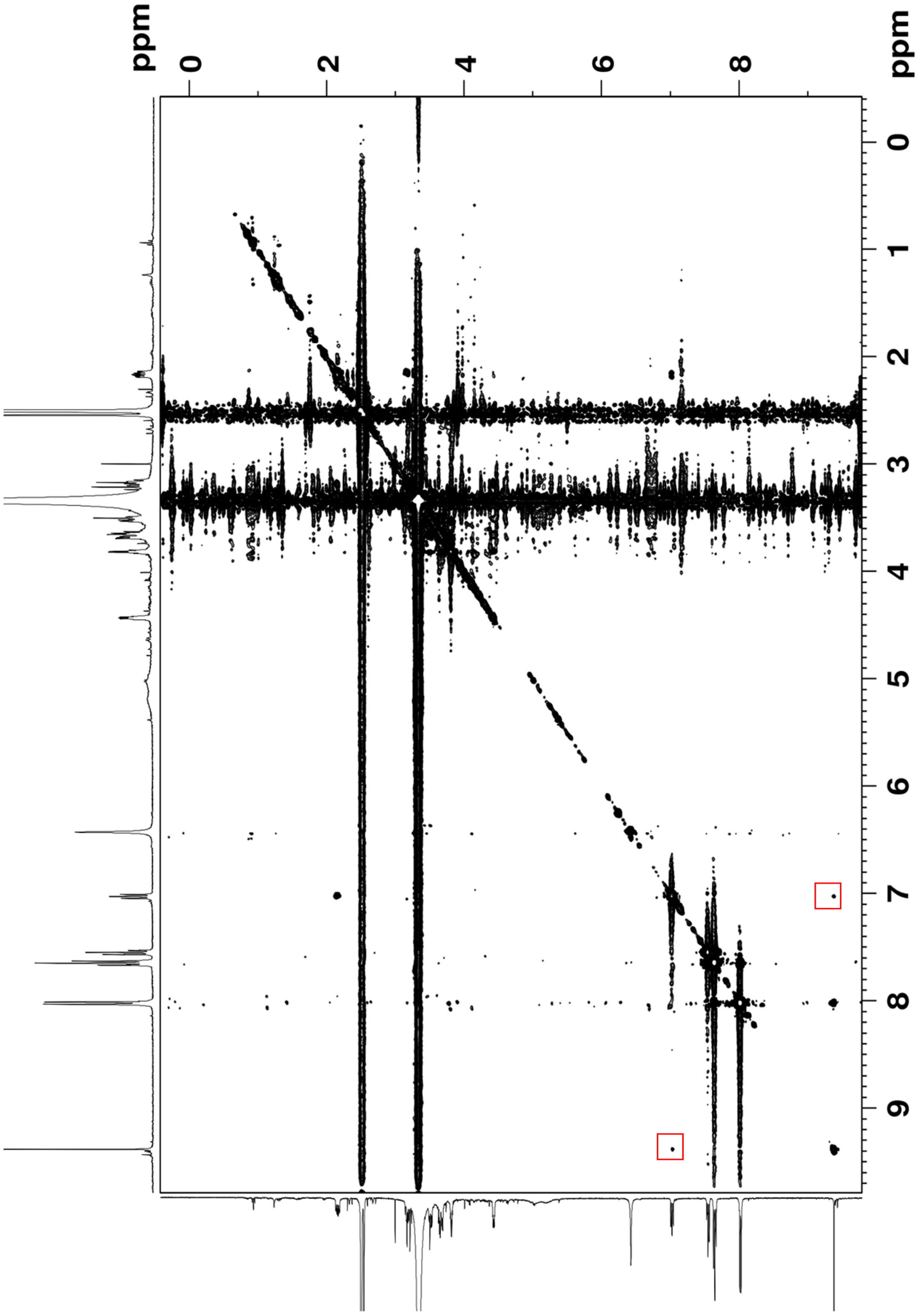

Figure S10. Full NOESY spectrum of 8^{et}dG click reaction product. Cross peak between H in the triazole ring and H1′ is in the red box.

ODN	Sequences	Calcd MS	Found MS
ODN-N	5'-TAGGGTTAGGGT-3'	3755.495	3754.836
ODN-3	5'-TA(8^{et}dG)GGTTAGGGT-3'	3779.495	3777.126
ODN-9	5'-TAGGGTTA(8^{et}dG)GGT-3'	3779.495	3777.313
Product(P)	5'-TAGGGTTA(P)GGT-3'	3898.545	3897.344

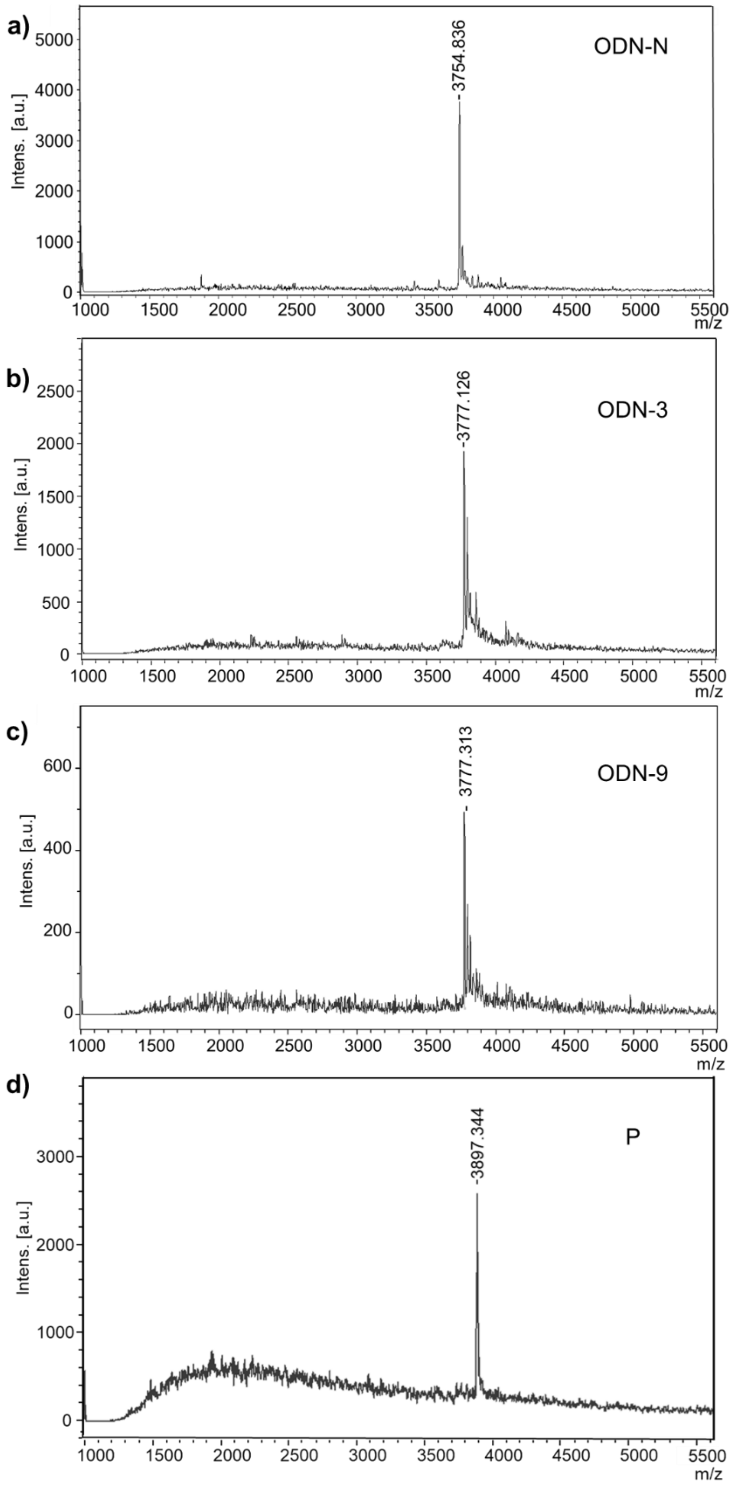

Figure S11 MALDI-TOF MS of ODNs used in this study.

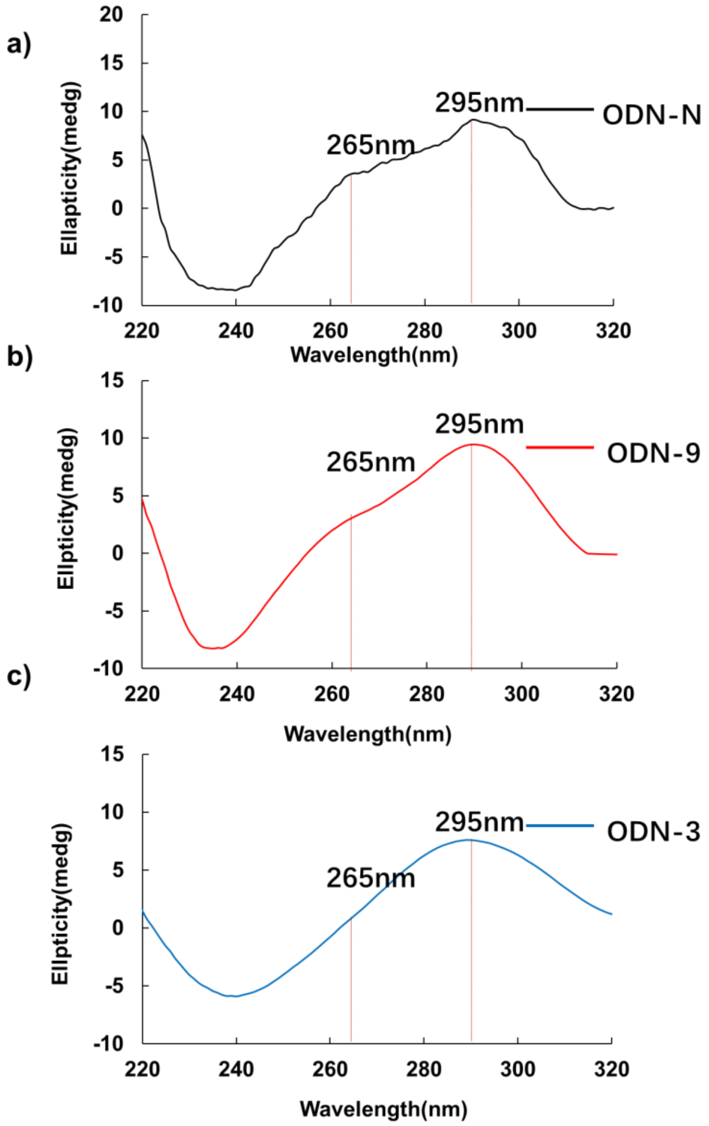

Figure S12 CD spectra of ODN-N(a);ODN-9(b);ODN-3(c) in the presence of 100 mM KCl at 25 °C. Both ODN-N and ODN-9 show positive peak at 265nm and 295nm.

1. Shinohara, Y.; Matsumoto, K.; Kugenuma, K.; Morii, T.; Saito, Y.; Saito, I. Design of environmentally sensitive fluorescent 2'-deoxyguanosine containing arylethynyl moieties: distinction of thymine base by base-discriminating fluorescent (BDF) probe. *Bioorganic & medicinal chemistry letters* **2010**, *20*, 2817-2820, doi:10.1016/j.bmcl.2010.03.055.
